# Consumer Brand Engagement Beyond the “Likes”

**DOI:** 10.3389/fpsyg.2021.692000

**Published:** 2021-09-23

**Authors:** Wiktor Razmus

**Affiliations:** Institute of Psychology, The John Paul II Catholic University of Lublin, Lublin, Poland

**Keywords:** consumer brand engagement (CBE), scale development, loyalty, satisfaction with product, perceived value of brand

## Abstract

In most consumer brand engagement (CBE) scales, indicators of CBE refer to behaviors that are related to social media or online brand communities. CBE also occurs beyond the Internet context in real-life settings. This paper reports the development and validation process of a CBE scale beyond the Internet behavior context. The results of three studies support the content validity, internal consistency, reliability, and nomological validity of the scale. Moreover, the results indicate that brand engagement measured by the CBE scale affects important aspects of brand-related consumer constructs. Consumers with a high level of brand engagement reflected greater brand loyalty, consumer satisfaction with a product, and perceived value of a brand. The author discusses the usefulness of this scale for marketing and psychological research.

## Introduction

Consumer brand engagement (CBE) is one of the key topics in research on marketing and consumer behaviors (Gómez-Suárez et al., 2017). This focus stems from the fact that the Marketing Science Institute (2010, 2014) included studies on consumer engagement among priority research directions for the future. Many studies regarding CBE explored how to define and measure CBE (Brodie et al., 2011; Hollebeek, 2011a; Vivek et al., 2012, 2014). One of the more popular CBE measurement instruments (with over 1,881 citations at the time of writing) is a scale proposed by Hollebeek et al. (2014). It is a valid measure with adequate psychometric properties, however it is applicable to social media settings. The same is true with other scales that focused on online communities or brand content in social media (Hollebeek et al., 2014; Dessart et al., 2016; Schivinski et al., 2016; Mirbagheri and Najmi, 2019). This means that indicators of CBE refer, for example, to reading posts in social media (*I read posts related to Brand X on social media*; Schivinski et al., 2016) or to participation in online community behavior (*I hope to improve the brand or product through my participation and expression in this brand community*; Baldus et al., 2015). These scales are very useful and provide a unique contribution relevant to a specific discipline and context. However, “the phenomenon of consumer engagement is not limited to the online environment” (Bilro and Loureiro, 2020b, p. 260). CBE also occurs beyond the Internet context in real-life settings and some people do not use social media for brand engagement behavior. While the above-mentioned brand engagement measures take into account the Internet as the interactive context of brand engagement, I argue that there is a substantial difference between measuring engagement with brands in online communities and social media and measuring engagement with brands beyond Internet behavior. A systematic review of customer engagement research in marketing showed that “there is a need to develop a much valid measure of customer engagement that can be generalized across multiple contexts” (Islam and Rahman, 2016, p. 2025). Recent analyses of existing scales demonstrated that CBE should be “measured more properly […] by means of situations which are more likely to happen in everyday life” (Ferreira et al., 2020, p. 501). This paper answers this call by developing a measure of CBE that is not limited to social media or other Internet behavior. The article offers two contributions to the literature on CBE. First, it presents the development of a CBE scale beyond the Internet behavior context that is based on the definition and model of this construct proposed by Hollebeek et al. (2014). Second, it highlights the value of the proposed scale not only by testing its structural and nomological validity but also by demonstrating in experimental research its potential for explaining marketing outcomes. The paper attempts to explore the nature of CBE and its marketing outcomes based on social exchange theory.

The remainder of this paper is organized as follows. It begins by analyzing a theoretical foundation of CBE and its conceptualization. Then, building on a review of the marketing and consumer psychology literature, a critical analysis of previous measurements of brand engagement is offered. Next, based on a theoretical framework and research findings, I propose hypotheses regarding the marketing consequences of CBE. The paper then develops and validates a scale for CBE, which is trying to condense three dimensions (cognitive, emotional, and behavioral) into a global measure that, consistently with scenario described by Rosado-Pinto and Loureiro (2020), can be employed in diverse brand contexts. The scale development and validation process comprised three studies that were conducted using five independent samples. After a standard procedure of item generation, scale purification, testing of nomological validity and test-retest reliability, I conducted an experiment to assess the validity of the scale. The final section of the paper offers a discussion, implications for theory and practice, limitations, and future research directions.

### Theoretical Foundation of Consumer Brand Engagement

Research on consumer engagement is primarily conducted using three theoretical backgrounds: (1) relationship marketing theory (Vivek et al., 2012); (2) service-dominant logic perspective (Brodie et al., 2011); and (3) social exchange theory (SET) (Hollebeek, 2011b). Relationship marketing theory and a service-dominant logic perspective have been utilized as a theoretical background for the marketing analysis of consumer and brand engagement. The well-established SET (Homans, 1958; Emerson, 1976) provides the opportunity to analyze CBE from a psychological perspective. According to this theory, consumers engage in interactions with others or objects (e.g., a brand) because they expect that their engagement will be rewarding (Emerson, 1976). For example, one side of the interaction (a brand) performs a “favor” (e.g., by providing the opportunity to signal high social status) for another side (a consumer) and then expects some future return (e.g., brand engagement and consumer loyalty). This partner relationship can be reversed: a consumer expects compensation for his or her positive thoughts and behaviors toward an object (a brand). With SET, exchange partners strive for balance in the relationship (Hollebeek, 2011b). Individuals engage in relationships based on a cost/benefit analysis and they remain in these relationships as long the benefits outweigh the costs. While economic exchange is based on tangible goods, social exchange involves both tangible and intangible rewards (information, pleasures of human contact, and social approval) (Homans, 1958; Chan and Li, 2010). SET is generally confined to exchanges of the same type of resource; however, an asymmetry in the resource exchange is also possible (Brinberg and Castell, 1982; Brinberg and Wood, 1983). According to Emerson (1976), such exchange is not limited to rational actions. “In place of calculation and reason in human affairs, it relies upon value as the result of prior conditioning in longitudinal exchange relationships” (Emerson, 1976, p. 341). As researchers have recently noted, the cost/reward perspective corresponds to the interactive nature of customer engagement (Hollebeek, 2011b). This alignment is consistent with the fundamental notion of SET, which claims that a series of interdependent transactions can produce attachment (Cropanzano and Mitchell, 2005).

### Conceptualization of Consumer Brand Engagement

CBE, despite being a fairly new construct in marketing (Brodie et al., 2011; Marbach et al., 2016), has received much attention from academic researchers (van Doorn et al., 2010; Brodie et al., 2011; Gambetti et al., 2012; Vivek et al., 2014; Dwivedi, 2015; Bilro and Loureiro, 2020a). However, its conceptualization stems from more general construct of consumer engagement, which definitions vary in the literature. For example, consumer engagement is defined as “a context-dependent, psychological state characterized by fluctuating intensity levels that occur within dynamic, iterative engagement processes” (Brodie et al., 2011, p. 260). By contrast, van Doorn et al. (2010, p. 254) considered consumer engagement from a behavioral perspective and defined it as “customer's behavioral manifestations that have a brand or firm focus, beyond purchase, resulting from motivational drivers.” A review of the various definitions (de Oliveira Santini et al., 2020) shows that consumer engagement can be defined as an intrinsic motivation concerning participation in brand community (Baldus et al., 2015), psychological mind state (Bowden, 2009; Brodie et al., 2011), consumer activities related to consumer–brand interactions (Hollebeek et al., 2014) or a customer's value addition to the firm (Pansari and Kumar, 2017). Therefore, CBE is a specific sub-form of consumer engagement, that occurs between the consumer(s) and the brand, which can operate online, offline or both (Bilro and Loureiro, 2020a).

Further, in the literature, considerable differences relate also to the dimensionality of CBE. Some studies describe CBE as a one-dimensional construct (Sprott et al., 2009), whereas others treat it as multidimensional (e.g., Dwivedi, 2015; Harrigan et al., 2018; see other examples in Table 1). The most recent research generally agrees that CBE should be operationalized as a multidimensional construct that captures cognitive, affective, and behavioral dimensions (e.g., Dessart et al., 2016; Leckie et al., 2016; Harrigan et al., 2018). In this paper I adopt the definition of CBE by Hollebeek et al. (2014), which is accepted by many researchers (e.g., Leckie et al., 2016; Harrigan et al., 2018). The authors define CBE as “a consumer's positively valenced brand-related cognitive, emotional and behavioral activity during or related to focal consumer/brand interactions” (Hollebeek et al., 2014, p. 154). Consistent with Hollebeek et al. (2014, p. 154), the cognitive dimension is defined as “a consumer's level of brand-related thought processing and elaboration in a particular consumer/brand interaction”; the emotional dimension is defined as “a consumer's degree of positive brand-related affect in a particular consumer/brand interaction”; and the behavioral dimension is described as “a consumer's level of energy, effort and time spent on a brand in a particular consumer/brand interaction.”

**Table 1:** Scales developed to measure brand engagement.

**References**	**Country**	**Construct**	**Context of brand engagement**	**Factors**	**Study details**	**Internal consistency**	**Test-retest reliability**	**Types of validity**
Sprott et al. (2009)	U.S.	Brand engagement in self-concept	offline context	Unidimensional	5 correlational studies 3 experimental studies	α = 0.94	*r* = 0.62–0.78	Content validityconstruct validity - structural - hypotheses testing
Hollebeek et al. (2014)	New Zealand	Consumer brand engagement in social media	Internet behavior context	Cognitive processing Affection Activation	1 qualitative study 3 correlational studies	α = 0.82–0.93	–	Content validityconstruct validity - structural - hypotheses testing
So et al. (2014)	Australia	Customer engagement with tourism brands	offline context	Identification Enthusiasm Attention Absorption Interaction	2 correlational studies	α = 0.86–0.94	–	content validityconstruct validity - structural - hypotheses testing
Vivek et al. (2014)	U.S.	Generalized customer engagement	Internet behavior and offline context	Conscious attention Enthused participation Social connection	4 qualitative studies 4 correlational studies	α = 0.83–0.96	–	Content validityconstruct validity - structural - hypotheses testing
Baldus et al. (2015)	U.S.	Online brand community engagement	Internet behavior context	Brand influence Brand passion Connecting Helping Like-minded discussion Rewards (hedonic) Rewards (utilitarian) Seeking assistance Self-expression Up-to-date information Validation	2 qualitative studies 4 correlational studies	α = 0.65–0.84	*r* =0.60	Content validityconstruct validity - structural - hypotheses testing
Dwivedi (2015)	Australia	Consumer brand engagement	offline context	Vigor Dedication Absorption	1 correlational study	α = 0.87–0.89	–	Construct validity- structural - hypotheses testing
Dessart et al. (2016)	UK France	Online brand communityengagement	Internet behavior context	*Second-order* Affective Cognitive Behavioral *First-order* Enthusiasm Enjoyment Attention Absorption Sharing Learning Endorsing	1 qualitative study 3 correlational studies	α = 0.90–0.98	–	Content validityconstruct validity - structural - hypotheses testing - cross-cultural validity
Dwivedi et al. (2016)	Australia	Brand engagement behaviors	Offline context	Interacting with other people Participating in marketing activities Collecting brand information	1 correlational study	CR = 0.92–0.94	–	Construct validity - structural - hypotheses testing
Schivinski et al. (2016)	Poland	Consumers' engagement with brand-related social-media content	Internet behavior context	Consumption Contribution Creation	3 qualitative studies 2 correlational studies	α = 0.88–0.93	–	Content validityconstruct validity - structural - hypotheses testing
Solem and Pedersen (2016)	Norway	Customer brand engagement in social media	Internet behavior context	Physical Emotional Cognitive	3 correlational studies	α = 0.81–0.93	–	Content validityconstruct validity - structural - hypotheses testing
Harrigan et al. (2017)	U.S.	Customer engagement with tourism social media brands	Internet behavior context	Absorption Identification Interaction	2 correlational studies	α = 0.87–0.95	–	Construct validity- structural - hypotheses testing
Harrigan et al. (2018)	U.S.	Consumer brand engagement	Internet behavior context	Cognitive processing Affection Activation	2 correlational studies	α = 0.85–0.93	–	Construct validity - structural - hypotheses testing
Obilo et al. (2021)	U.S.	Consumer brand engagement in social media	Internet behavior context	Content engagement Co-creation Advocacy Negative engagement	2 correlational studies	CR > 0.70	–	Construct validity - structural

To understand the concept of CBE it is necessary to differentiate it from other similar marketing constructs, for example, consumer brand involvement, brand attachment, self-brand connection, and brand love. Consumer brand involvement is defined as a person's perceived relevance of the brand based on inherent needs, values, and interests (Zaichkowsky, 1985). Brand involvement includes cognitive, affective, and motivational dimensions, whereas CBE involves attitudinal as well as behavioral responses toward a brand (Parihar et al., 2018). Involvement occurs due to the interest level while highly engaged consumers invest thoughts, emotions, and behaviors due to a felt connection with the brand (Harrigan et al., 2018). Consumer brand involvement is a predictor of CBE (Hollebeek et al., 2014; Harrigan et al., 2018). Other constructs similar to CBE are brand attachment and brand love. The former is defined as “the strength of the bond connecting the brand with the self […], that involves thoughts and feelings about the brand and the brand's relationship to the self” (Whan Park et al., 2010, p. 2), while the latter is considered as a relationship between consumer and brand(s) that involves long-lasting and deep feeling for the brand (Langner et al., 2015). Brand attachment and brand love are, above all, emotion-laden target-specific bonds between a consumer and brand(s) (Thomson et al., 2005; Loureiro et al., 2017; Verma, 2021), whereas CBE goes beyond the mere emotional aspect, taking into account also the cognitive and behavioral aspects (Hollebeek et al., 2014). And finally, self-brand connection is treated as the extent to which individuals incorporate the brand(s) into their self-concept (Escalas and Bettman, 2003). CBE does not assume that consumers linked brand(s) to the self and, as research showed, self-brand connection should be viewed rather as a consequence of CBE (e.g., Hollebeek et al., 2014).

The relationship between CBE and other marketing constructs that are not described here are discussed, for example, by Hollebeek (2011a) or Brodie et al. (2011).

### Previous Measurement of Brand Engagement

Analyses of brand engagement are performed in two main areas: (1) engagement in virtual aspects of the brand, such as online communities or brand content in social media (Karpińska-Krakowiak, 2014; Dessart et al., 2016; Schivinski et al., 2016) and (2) engagement outside the Internet behavior context (Sprott et al., 2009; Razmus et al., 2017). Several measures have been developed to assess brand engagement. To contribute to an analysis of previous scales to measure this construct, I performed a literature search and identified English peer-reviewed articles related to this issue. The following electronic databases were employed: Scopus and Web of Science. “Brand engagement scale” in titles, keywords or abstracts was searched. The research produced 243 records, including duplicates (at the end of June 2021). I identified numerous articles related to diverse engagement foci. Taking into account that consumer engagement is context-dependent (Hollebeek, 2011a), the analysis focused on articles that strictly concerned “brand” engagement. After the rejection of duplicates and selecting those that focused on scale development, 13 papers were considered for review (Table 1). Evaluation of the psychometric properties of existing scales was performed using the Consensus-based Standards for the selection of health Measurement Instruments (COSMIN) checklist (Mokkink et al., 2010), which is a well-developed framework for evaluating measures. I focused on two aspects of reliability – internal consistency and test-retest reliability – and three aspects of validity – content validity, construct validity (structural validity, hypotheses testing, cross-cultural validity) and criterion validity. A summary of the existing scales is provided in Table 1.

Prior research that explores connections between consumers and their brands defined these connections as self–brand connections (Escalas and Bettman, 2003). The first scale for measuring brand engagement, introducing a new construct (brand engagement in self-concept), was developed by Sprott et al. (2009). These researchers focused on a specific understanding of engagement as a generalized propensity to include important brands as part of the self-concept. The first conceptualization of the “consumer brand engagement” construct without referring it to the self was proposed by Hollebeek et al. (2014), who defined CBE as “a consumer's positively valenced brand-related cognitive, emotional and behavioral activity during or related to focal consumer/brand interactions” (Hollebeek et al., 2014, p. 154). The 10-item CBE scale that was developed by the authors comprised three factors and its validity was confirmed in three correlational studies. It should be noted that the construct proposed by Hollebeek et al. (2014) refers to engagement in social media (in validation studies, they measured consumer engagement with Twitter and Facebook as interactive brands). Since 2014, several scales for brand engagement measurement in the Internet behavior context have been created. In the U.S., a scale for the assessment of online brand community engagement was developed by Baldus et al. (2015). One year later, a scale for measuring the same construct was proposed in the UK by Dessart et al. (2016). Other scales related to brand engagement measurement in the Internet behavior context are presented in Table 1. Generally, nine scales exist for brand engagement measurement in the Internet behavior context (one scale can be applied to the Internet and offline context). Because they are not the main subjects of the paper, I will focus on scales that measure brand engagement based on indicators that refer to real-life settings (beyond the Internet behavior context).

Four different scales were proposed by scholars to capture brand engagement beyond the Internet behavior context (Table 1). So et al. (2014) developed the scale of customer engagement with tourism brands in two correlational studies. The scale comprehended five factors—Identification, Enthusiasm, Attention, Absorption, and Interaction—and had adequate psychometric properties (adequate internal consistency and nomological validity). A different approach to CBE scale development was proposed by Dwivedi (2015). He adapted the concept of employee engagement and examined its factorial validity in a consumer–brand relationship context. The results of one correlational study supported a three-factor structure of CBE (Vigor, Dedication, Absorption) with adequate internal consistency and nomological validity. Dwivedi et al. (2016) examined Keller's Actual Brand Engagement framework and validated the brand engagement behaviors scale. A three-factor structure, with internal consistency and nomological, convergent, and discriminant validity of the scale, was proven in one correlational study. The scale of generalized customer engagement, which consists of three factors (Conscious Attention, Enthused Participation and Social Connection) and can be applicable across several contexts (brand and retail), was proposed by Vivek et al. (2014). The internal consistency and nomological validity of the scale were indicated in four correlational studies.

Summing up, most scales for brand engagement measurement refer to the Internet behavior context (social media or online brand community), whereas scales that examine brand engagement in an offline context have limitations. First, the validity of the scale developed by Dwivedi (2015) is problematic because it was adapted from organizational psychology. Indicators of brand engagement in the scale are derived from a different context and their meaning may not be appropriate in the context of brands (e.g., *when I get up in the morning, I feel like using my mobile*). This may explain why some researchers attempt to combine two scales (the scale proposed by Dwivedi and another) into one scale (Fernandes and Moreira, 2019). Second, for all four scales that can be used to measure brand engagement beyond the Internet behavior context, the possibility of their use for brands in different product categories is problematic. The scale proposed by So et al. (2014) was created to measure engagement with tourism brands and has not been validated in other product contexts. In a validation study of a generalized multidimensional scale of customer engagement (Vivek et al., 2014), only one brand (Apple) was employed, and single product brands (mobile phone and tablet, respectively) were also applied in the studies conducted by Dwivedi (2015) and Dwivedi et al. (2016). Marketing studies should employ a representative sample of stimuli (in this case, brands and products) in addition to a representative sample of subjects that prove the nomothetic nature of studies and enable broader conclusions to be obtained. Recent analyses of Vivek et al.'s scale showed that it was also inefficient in discriminating weakly and strongly engaged individuals (Ferreira et al., 2020). The brand engagement behaviors scale (Dwivedi et al., 2016) focused on the behavioral aspect of brand engagement. This solution reduces the understanding of brand engagement and omits other important aspects of the construct. These limitations of the scales corroborate with previous calls (Islam and Rahman, 2016) to develop a considerably more valid scale that satisfies development procedures, both from a conceptual and a methodological standpoint. In the next part of the paper, I will present such a proposal.

### Consumer Brand Engagement and Its Marketing Consequences

**Figure 1:**
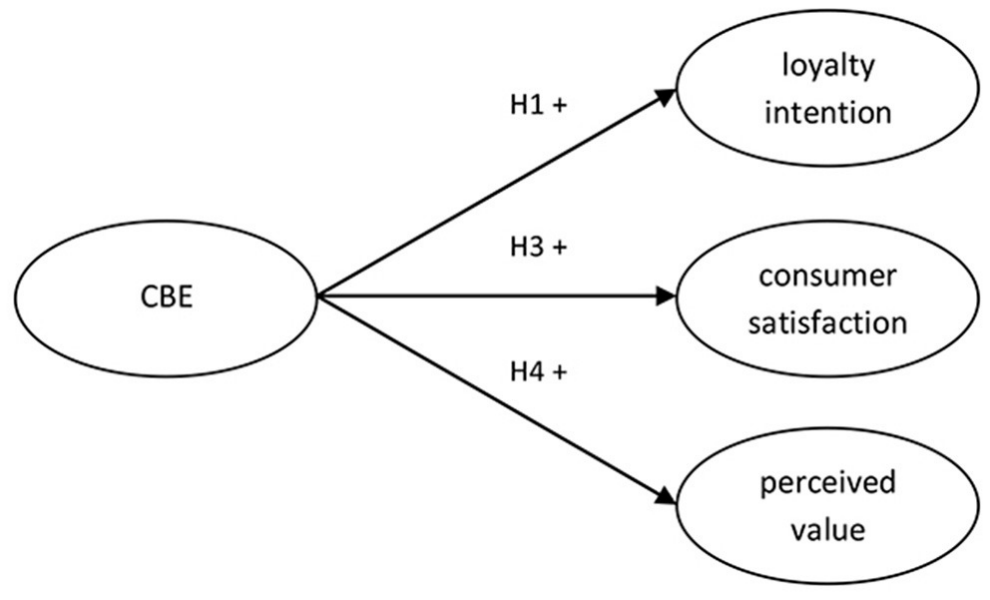
Nomological net of selected CBE conceptual relationships.

A review of the literature indicates that CBE is an important variable that affects diverse marketing consequences (Islam and Rahman, 2016; Rosado-Pinto and Loureiro, 2020). In current research, I outline three outcomes of CBE: loyalty intention, consumer satisfaction with product, and perceived value of brand. In this section, I will develop hypotheses regarding the marketing consequences of CBE (Figure 1).

Loyalty intention is a customer's intention to say positive things about a brand, recommend a brand to other people, and declare to purchase this brand in the future (Zeithaml et al., 1996). Hollebeek argues that CBE is a relational construct and the research results indicate that CBE causes the formation of psychological bonds with a brand (Hollebeek et al., 2014; Harrigan et al., 2018; Tunca, 2019). Highly engaged consumers invest thoughts, emotions, and behaviors into their preferred brands and, according to SET (Emerson, 1976), they in turn receive valuable resources from these brands. Consumers are likely to commit to preserving this relationship and loyalty may be the mechanism that regulates it (Dwivedi, 2015). Evidence supports that CBE is a predictor of loyalty in the Internet and offline context (Dwivedi, 2015; Islam and Rahman, 2016; Harrigan et al., 2017). Based on a theoretical framework and research findings, I hypothesize:

*Hypothesis 1:* CBE is positively related to loyalty intention.

However, although CBE is expected to predict loyalty intention (Dwivedi, 2015; Islam and Rahman, 2016; Harrigan et al., 2017), the literature focuses mainly on correlational research. I want to verify the relationship between CBE and loyalty intention also in an experimental approach and treat CBE as a moderator. This analysis will expand correlational study findings and provide insight that explains how CBE influences brand loyalty. As follows from the definition of loyalty proposed by Zeithaml et al. (1996), loyalty encompasses not only repeated purchases. There is research on brand engagement-related constructs and such aspects of brand loyalty as positive attitude toward brand and brand advocacy or time insensitivity. For example, a series of experiments conducted by Lisjak et al. (2012) provided evidence that individuals who identify with a brand defend it when the brand is threatened (i.e., when negative massages concerning the brand are formulated). Another experimental study showed that consumers with a high level of brand engagement in self-concept are willing to wait longer for a new offering from their favorite brand than consumers with a low level of this variable (Sprott et al., 2009). Based on these research findings, I focus on time sensitivity regarding a delay in a brand's new product introduction. Because CBE generates positive attitudes toward brands (Vivek et al., 2014) and leads to intense relational bonds with a brand (Dwivedi, 2015), consumers with high level of CBE should be willing to wait longer for a new product introduced by their favorite brand. Based on this, it was hypothesized:

*Hypothesis 2:* CBE moderates the relationship between waiting time and willingness to wait for a person's favorite brand: in low-CBE individuals, longer waiting times reduce the willingness to wait for a product of the favorite brand, whereas in high-CBE individuals the willingness to wait does not change depending on the waiting time.

Consumer satisfaction is treated as “a global evaluative judgment about product usage/consumption” (Westbrook, 1987, p. 260). Customer engagement in the relationship with brands enables greater satisfaction due to their personal investment in the brand. It is conceivable to assume that individuals with a high level of CBE may be satisfied with the brand. In early theoretical analyses of CBE, satisfaction has been considered as an engagement consequence (Brodie et al., 2011; Hollebeek, 2011a) and this assumption has been confirmed in research. The association between brand engagement and satisfaction is significantly positive in the case of both functional and emotional brands (Fernandes and Moreira, 2019). Consumers engaged with social media brand communities tend to exhibit higher levels of satisfaction (Brodie et al., 2013; Carvalho and Fernandes, 2018). Therefore, the following hypothesis is postulated:

*Hypothesis 3:* CBE is positively related to consumer satisfaction with the product.

Perceived value is a construct that “encompasses perceptions of quality given price and inputs vs. outputs relative to the competition” (Johnson et al., 2006, p. 123). In consumer behavior literature, researchers treated perceived value as antecedents of consumer/brand engagement (Chen, 2017; Leckie et al., 2018). Previous empirical studies also demonstrated that perceived value can be treated as a consequence of consumer/brand engagement. This approach is consistent with early theoretical analyses of customer engagement. For example, Vivek et al. (2012, p. 134) stressed that “a highly engaged individual will derive both intrinsic and extrinsic value from his or her focus of engagement.” Research shows that engagement with mobile social networks positively influences perceived advertising value (Wu, 2016). The findings of past empirical research also imply a positive relationship between online consumer engagement and customer-perceived value (Marbach et al., 2016). Based on these rationales, I propose the following hypothesis:

*Hypothesis 4:* CBE is positively related to the perceived value of the brand.

## Scale Development and Validation Process

The aim of current studies, as described here, was to develop a scale for CBE that is treated as a multifaceted construct comprising three dimensions: cognitive, emotional, and behavioral (Hollebeek et al., 2014). A second-order construct is suggested, where the three dimensions collectively represent a more abstract construct of CBE. Studies on other brand engagement scales based on a three-dimensional structure (cognitive, emotional, and behavioral) reported high positive correlations among the scales (Hollebeek et al., 2014; Tunca, 2019; Ferreira et al., 2020). In this case, a second-order model is recommended (Chen et al., 2006), as employed and supported in many brand engagement scale developments (So et al., 2014; Dwivedi, 2015; Dessart et al., 2016; Dwivedi et al., 2016). Scale development and validation proceeded in three main stages. In the first stage, an initial item pool was created and purified. Within this stage, a qualitative study (Study 1, *N* = 30) was conducted to identify consumers' natural descriptions regarding brand engagement behavior. Next, a scale purification study (Study 1, *N* = 417) was carried out to reduce the number of items and select those with the highest psychometric properties. In this stage, I also tested the nomological validity, which is the predictive validity of the scale with regard to other constructs related to consumer behavior. The aim of the second stage was to further analyze the structure of the CBE scale in another sample of individuals (Study 2, *N* = 339) and investigate the test-retest reliability (Study 2, *N* = 151). In the third and final stage, I conducted one experiment (Study 3, *N* = 98) to analyze the validity of the CBE scale. The aim of this study was to check if brand engagement measured by the CBE scale affects the indicator of brand loyalty.

## Study 1: Brand Engagement Indicators, Item Purification, and Nomological Validity of the CBE Scale

The purpose of Study 1 was to: find out what indicators of brand engagement beyond the Internet context are listed by consumers; develop a new scale; and examine its psychometric properties and nomological validity.

### Participants

This study involved two samples. The first sample consisted of 30 individuals (15 women) whose age ranged from 17 to 54 years (*M* = 29.00, *SD* = 9.40). Approximately half of the participants lived in cities with more than 100,000 inhabitants (46.7%), 36.7% lived in cities with a maximum of 100,000 inhabitants, and 16.6% lived in the countryside. Participants from this sample took part in structured face-to-face individual interviews that were aimed at identifying the consumers' natural descriptions regarding brand engagement behavior.

The second sample consisted of 417 individuals (231 women) aged 17–62 years (*M* = 25.20, *SD* = 6.33). Close to half of the participants lived in cities with more than 100,000 inhabitants (49.2%), 23.9% lived in cities with a maximum of 100,000 inhabitants, and 26.8% lived in the countryside.

### Brand Engagement Indicators—The Structured Face-To-Face Individual Interviews

Structured individual interviews with brand-engaged consumers were conducted in one-to-one settings. The participants were recruited from a variety of places (e.g., universities, organizations, neighborhoods) using a convenience sampling method. It was assumed that brand-engaged consumers should satisfy two criteria: have their favorite brand and declare to undertake behavioral, emotional or cognitive behavior toward their favorite brand when they discover that the products of their brand are no longer available on the market. The interviews were conducted in Polish and consisted of two parts. First, respondents were asked to answer the following open question: How do you define *engagement*? Second, respondents were asked to think about a brand with which they are engaged and another brand with which they do not feel engaged. In this part of the interview, the following open questions were asked: *How do you manifest your brand engagement? What behaviors, emotions and thoughts are related to your favorite brand and which of these are not related to the non-engaging brand?* I also asked the respondents questions related to the cognitive, emotional and behavioral facets of brand engagement sourced from the literature review. The interviews were audio-recorded and then transcribed. Participation in the study was entirely voluntary without financial incentive.

### Brand Engagement Indicators and Item Generation

Respondents who were engaged in their favorite brands have indicated many aspects of cognitive, emotional, and behavioral engagement. Two researchers conducted content analysis and categorized the available data (Lindlof and Taylor, 2010). They read through all of the interview transcripts, independently summarized the interview data into a set of themes and then worked together by discussing and improving the themes to ensure that consumers' statements were accurately understood. The results revealed five indicators of cognitive brand engagement: (1) positive memories about the brand; (2) thinking about the brand; (3) treating the brand as one's own; (4) paying attention to the message related to the brand; and (5) following novelties related to the brand. Researchers also revealed six main indicators of emotional brand engagement: (1) sense of pride in having and using the products of one's favorite brand; (2) feeling of pleasure in having and using the products of one's favorite brand; (3) feeling of joy in having and using the products of one's favorite brand; (4) lack of strong negative emotions when buying a defective product from this brand; (5) feeling negative emotions in a situation in which the consumer cannot purchase the product of his or her favorite brand; and (6) feeling negative emotions when someone criticizes their favorite brand. In the case of behavioral indicators, researchers revealed the following: (1) the need to advertise the brand by visibly using it; (2) taking extra effort to obtain the product of one's favorite brand; (3) following information about the brand actively; (4) a tendency to recommend the brand to friends; (5) defending the brand when others criticize it; and (6) talking about the brand with others. Examples of consumer statements are presented in Appendix 1. Based on the results of interviews that enabled a deeper understanding of brand engagement, two researchers (those who did the content analysis) generated initial items. The next step involved reviewing and editing items to eliminate redundant items and select these that had good content as well as face validity. Two other experts evaluated how well each item represented CBE. Items considered to be inapplicable were removed, and after this procedure a pool of 21 items was obtained. The items were checked by a Polish language expert for clarity of the language and some of them were revised.

### Item Purification and Nomological Validity Testing

The aim of this stage was to evaluate the dimensionality of the construct, reduce the 21-item pool, which reflects brand engagement, and test the nomological validity of the CBE scale.

### Measures

#### Consumer Brand Engagement

Twenty-one items derived from the initial qualitative study to measure brand engagement were used. Each subscale (cognitive, emotional and behavioral) includes seven items rated on a five-point scale from 1 (*strongly disagree*) to 5 (*strongly agree*).

#### Loyalty Intention

Brand loyalty was measured with three items from the Loyalty Intentions Scale (items 1–3; Johnson et al., 2006). Items (e.g., *Next time, I will definitely buy this brand again; If I lose my product, I will definitely buy it again*) were rated on a seven-point scale from 1 (*strongly disagree*) to 7 (*strongly agree*). In this study, Cronbach's α was 0.69.

#### Consumer Satisfaction

The three-item Customer Satisfaction Scale (Homburg et al., 2012) was applied. Items (e.g., *This [product] totally meets my expectations; All in all, I am very satisfied with the [product]*) were rated on a seven-point scale from 1 (*totally disagree*) to 7 (*totally agree*). In this study, Cronbach's α was 0.90.

#### Perceived Value

Participants completed the Perceived Value Scale (Johnson et al., 2006), which consists of four items (e.g., *Product of this brand is a good level of performance for the money I pay; Product of this brand is a great value*). Items were rated on a seven-point scale from 1 (*totally disagree*) to 7 (*totally agree*). In this study, Cronbach's α was 0.78.

### Procedure

Data were collected through online surveys (Qualtrics software) by four research assistants. Participants were recruited by direct solicitation of the data collectors, who went into various venues or contacted individuals *via* social media, described the study and encouraged them to participate. Before participants completed the online version of the questionnaires, they provided informed consent. The respondents chose one of ten products (cars, mobile phones, RTV equipment, computer equipment, clothes, coffee, shoes, cosmetics, perfumes, and chocolate), among which was their favorite brand. Then, the respondents chose the brand that they liked and completed the scales. To reduce the chance of socially desirable responses, I provided the general topic of the survey and ensured the respondents' anonymity. Participation in the study was voluntary and the respondents did not receive any reward. To minimize the incidence of common method bias, I divided the questionnaire into sections, and items related to CBE and outcome constructs were presented on separate pages.

### Statistical Analyses

Because the aim was to construct the scale based on the existing brand engagement definition and model (Hollebeek et al., 2014), Confirmatory Factor Analysis (CFA) using Mplus v.7.0 (Muthén and Muthén, 2012) was performed. Considering the multivariate non-normality estimate, models were tested using maximum likelihood estimation with robust standard errors. The following indices were taken into account when assessing the model fit: Satorra-Bentler Scaled Chi-Square (S-Bχ^2^), Root Mean Square Error of Approximation (RMSEA), Comparative Fit Index (CFI), and Standardized Root Mean Squared Residual (SRMR). RMSEA and SRMR values below 0.08 and CFI values higher than 0.90 indicate an acceptable fit (Brown, 2006). To test the nomological validity of the CBE scale, I employed structural equation modeling. The convergent validity of the model was investigated by examining Average Variance Extracted (AVE) and Composite Reliability (CR). This kind of validity is achieved when AVE for every construct exceeds 0.50 and CR exceeds 0.70. I also tested the discriminant validity of the model. This validity is achieved when the square root of AVE for the construct is higher than its correlations with other constructs (Hair et al., 2009).

## Results

### Preliminary Analyses

A maximum of 0.5% data were missing completely at random in the items [Little's MCAR test: ${\text{χ}}_{\left(28\right)}^{2}$ = 37.832, *p* < 0.102]. Respondents described brands from all product categories (from 5.8% of computer brands to 12.9% of car brands). Harman's single factor test was employed to assess the common method variance (Podsakoff et al., 2003). An exploratory factor analysis (EFA) of all items with one factor and an unrotated solution showed that this factor explained ~28% of the variance in all variables. This result means that the collected data are essentially free from common method bias.

### CBE Scale Structure

**Figure 2:**
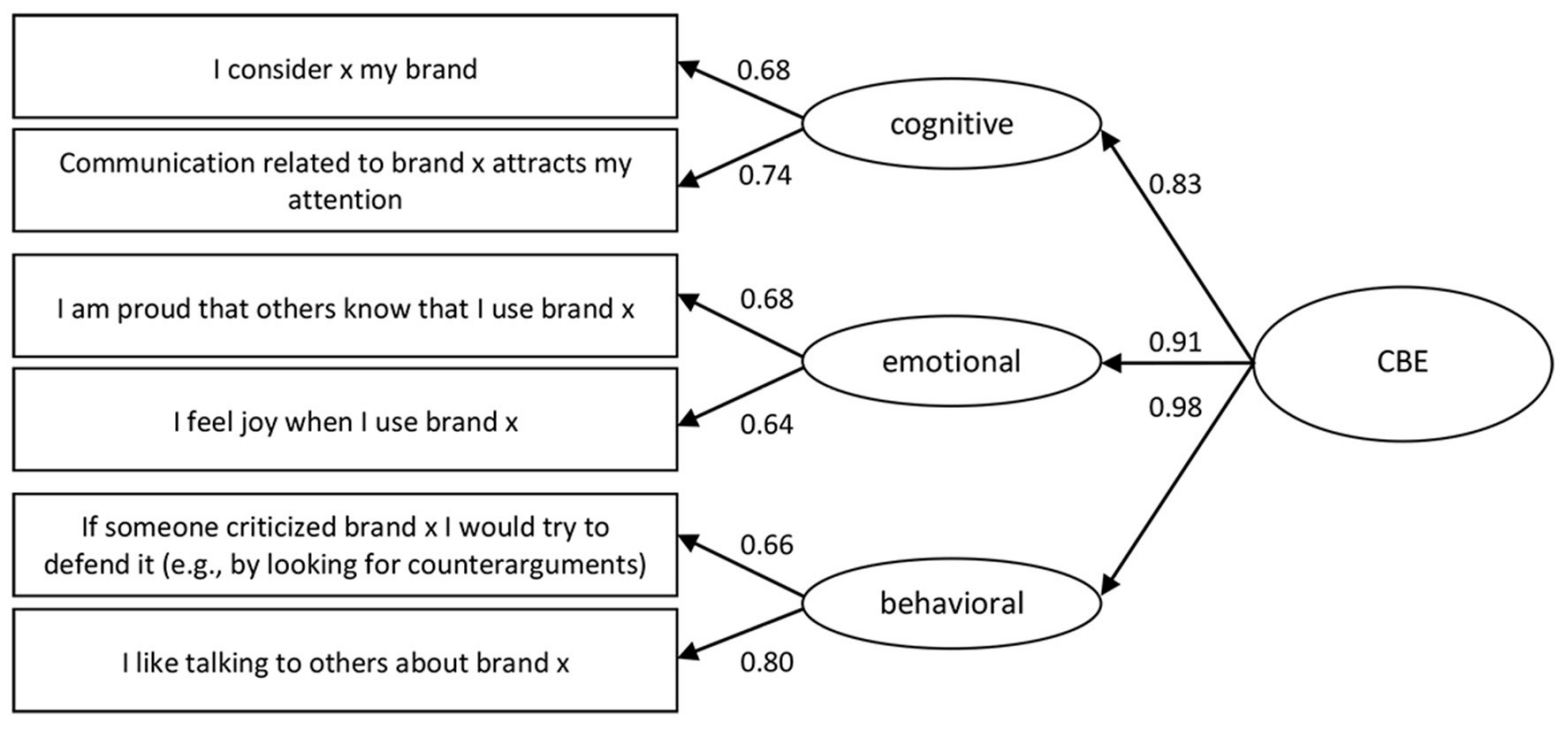
The higher order model of CBE (Study 1).

In the first step, two items for each dimension were removed from the scale based on item-total correlation values (cut-off values < 0.40). In the second step, a hierarchical CFA with three first-order factors was performed. A model with five items in each dimension had an unacceptable model fit: S-B${\text{χ}}_{\left(87\right)}^{2}$ = 566.815, *p* < 0.001, RMSEA = 0.115 with 90% CI = 0.106–0.124, CFI = 0.756, SRMR = 0.077. Items with low factor loadings (below 0.50) were removed, and the model with two items in each dimension showed acceptable fit indices: S-B${\text{χ}}_{\left(6\right)}^{2}$ = 11.177, *p* < 0.083, RMSEA = 0.045 with 90% CI = 0.000–0.086, CFI = 0.991, SRMR = 0.019. Factor loadings for the first-order factors ranged from 0.64 to 0.80 (Table 2), and factor loadings for the second-order factors range from 0.83 to 0.98 (Figure 2). Cronbach's α for the entire scale was 0.81 and item-total correlations ranged from 0.54 to 0.67 (Table 2).

**Table 2:** The CBE items with factor loadings, item-total correlations, means and standard deviations (Study 1).

**Dimensions**	**Items**	**Factor loadings**	**Item-total correlations**	***M***	***SD***
Cognitive	I consider x my brand	0.68	0.54	3.32	1.08
	Communication related to brand x attracts my attention	0.74	0.57	3.19	1.09
Emotional	I am proud that others know that I use brand x	0.68	0.56	3.24	1.12
	I feel joy when I use brand x	0.64	0.54	3.89	0.88
Behavioral	If someone criticized brand x I would try to defend it (e.g., by looking for counterarguments)	0.66	0.56	3.00	1.04
	I like talking to others about brand x	0.80	0.67	3.11	1.08

### Nomological Validity of CBE Scale

First, the CFA of the measurement model was tested. In this model, I specified the posited relationships of the observed indicators to the latent constructs (CBE and marketing consequences) and allowed all constructs to be inter-correlated. The model was well-fitted to the data: S-B${\text{χ}}_{\left(95\right)}^{2}$ = 229.257, *p* < 0.001, RMSEA = 0.058 with 90% CI = 0.049–0.068, CFI = 0.941, SRMR = 0.061. Then, the convergent and discriminant validity was investigated. Table 3 presents the results, which provided evidence of adequate convergent and discriminant validity of the scales (with the exception of AVE = 0.49 for the perceived value measure, which was marginally lower than required).

**Table 3:** The convergent and discriminant validity indices of the measurement model.

**Construct**	**CR**	**AVE**	**Correlation of constructs and average variance extracted**			
			**(1)**	**(2)**	**(3)**	**(4)**
(1) Consumer satisfaction	0.90	0.75	**0.87**			
(2) CBE	0.94	0.83	0.48	**0.91**		
(3) Perceived value	0.79	0.49	0.58	0.51	**0.70**	
(4) Loyalty intention	0.75	0.52	0.63	0.46	0.58	**0.72**

*The diagonal values (in bold) are the square roots of AVE of the constructs*.

Second, I tested the structural model, in which covariances between latent variables (from CBE and its consequences) were converted into regression paths implied by the assumed structure of the model. CBE was positively related to loyalty intention (β = 0.46, *p* < 0.001, *R*^2^ = 0.21), consumer satisfaction with product (β = 0.48, *p* < 0.001, *R*^2^ = 0.23), and perceived value of brand (β = 0.51, *p* < 0.001, *R*^2^ = 0.26), thus Hypotheses 1, 3, and 4 were supported.

## Study 2: Further Analysis of the CBE Scale Structure and Test-Retest Reliability

The aim of Study 2 was to further analyze the structure of the CBE scale in another sample of individuals and investigate the scale's test-retest reliability.

### Participants

The study involved two subsamples. Data from the first subsample were used to further examine the factor structure of the CBE scale. This subsample consisted of 339 individuals (176 women) whose age ranged from 18 to 57 years (*M* = 35.35, *SD* = 11.12). More than half of the participants lived in cities with over 100,000 inhabitants (65.4%), 23.0% lived in cities with a maximum of 100,000 inhabitants, and 11.5% lived in the countryside.

Data from the second subsample were used to investigate the test-retest reliability. The second subsample consisted of 151 individuals (74 women) aged 16–38 years (*M* = 26.58, *SD* = 5.53). Approximately one third of the participants lived in cities with over 100,000 inhabitants (33.5%), 43.0% lived in cities with a maximum of 100,000 inhabitants, and 23.5% lived in the countryside.

### Measures

#### Consumer Brand Engagement

The CBE scale developed in Study 1 (Table 2) was employed. Answers for six items were provided on a five-point scale from 1 (*strongly disagree*) to 5 (*strongly agree*). An average score was computed for each participant, where higher scores represent a high level of CBE.

### Procedure

Data were collected using paper-and-pencil questionnaires at participants' homes or work sites. Individuals were visited by research assistants who delivered and collected completed questionnaires. All respondents were selected by convenience sampling and recruited *via* personal contacts of the data collectors. The participants provided informed consent before they completed anonymous questionnaires. In the case of the test-retest procedure, respondents were contacted and asked to complete the CBE scale for a second time 2 weeks after the first measurement. Participants from the first subsample (*N* = 339) assessed their engagement in the clothing brand they chose, while participants from the second subsample (*N* = 151) evaluated their engagement in brand from various product categories. Participation in the study was voluntary and the respondents did not receive any reward.

### Statistical Analyses

CFA using Mplus v.7.0 (Muthén and Muthén, 2012) was performed to test the factor structure of the CBE scale. The procedures for assessing the model fit, as described in Study 1, were followed. For the test-retest sample, intraclass correlation coefficients and paired-sample *t*-tests were used to estimate the stability of the CBE scale scores in women and men separately.

## Results

### Preliminary Analyses

There were no missing data in CBE scale scores in the first and second subsamples.

### The CBE Scale Structure

The second-order factor model showed good fit indices: S-B${\text{χ}}_{\left(6\right)}^{2}$ = 9.218, *p* < 0.161, RMSEA = 0.040 with 90% CI = 0.000–0.088, CFI = 0.993, SRMR = 0.023. Factor loadings for the first-order factors ranged from 0.55 to 0.83, and factor loadings for the second-order factor exceeded 0.82. Cronbach's α was 0.83 and the item-total correlations ranged from 0.48 to 0.69.

### Test-Retest Reliability

In the female subsample, the intraclass correlation coefficient between the CBE scores from the first measurement and those from the second measurement was 0.85, with 90% CI = 0.76–0.91. There were no statistically significant differences between the first (*M* = 21.27, *SD* = 4.34) and the second measurement (*M* = 21.14, *SD* = 4.23): *t*_(73)_ = 0.50 and *p* < 0.691. In the male subsample, the intraclass correlation coefficient between the CBE scores from the first measurement and those from the second measurement was 0.86, with 90% CI = 0.78–0.92. Mean scores were not significantly different across the first (*M* = 21.01, *SD* = 4.78) and the second measurement (*M* = 21.06, *SD* = 5.08): *t*_(76)_ = −0.17 and *p* < 0.862. The current study has confirmed the test-retest reliability of the CBE scale.

## Study 3: CBE and the Impact of Waiting Time on Willingness to Wait for a Person's Favorite Brand

The aim of Study 3 was to check if brand engagement measured by the CBE scale affects the indicator of brand loyalty.

### Participants

Participants of the study comprised 98 individuals (50 women) whose age ranged from 19 to 39 years (*M* = 26.04, *SD* = 4.96). Most participants lived in cities with more than 100,000 inhabitants (57.7%), 24.7% lived in cities with a maximum of 100,000 inhabitants, and 17.5% lived in the countryside.

### Measures

#### Consumer Brand Engagement

The CBE scale developed in Study 1 was used. In the current study, Cronbach's α was 0.72.

#### Preference to Wait

The willingness to wait was measured with an 11-point scale from 1 (*I will not wait and buy the currently available brand now – 0% chance*) to 11 (*I will definitely wait and buy my favorite brand – 100% chance*).

### Procedure

Respondents were invited to take part in a short experimental study. All respondents were selected by convenience sampling and recruited *via* personal contacts of the data collectors. The study was conducted by two research assistants who visited participants at their homes or work sites. Individuals took part in the study using a computer (Qualtrics software) provided by data collectors. Prior to the experiment, participants gave informed consent. At the beginning of the study, the subjects were asked to provide their favorite smartphone brand. The name of this brand appeared in further questions. Each participant was asked to imagine a situation in which he or she considers the purchase of a new smartphone (a commonly used product nowadays). Next, they read a piece of information about a new model of smartphone for which they had to wait (scenario is available upon request). The experimental manipulation consisted of specifying the waiting time for the new smartphone model. In the first group (*N* = 57) the waiting time was 1 month and in the second group (*N* = 41) it was 6 months. Next, the participants were asked to answer the question concerning their willingness to wait for the product of their favorite brand. Participation in the study was voluntary and the respondents did not receive any reward.

### Statistical Analyses

To examine a moderation analysis, Hayes' PROCESS macro v.3.4 (Model 1; Hayes, 2013) was utilized. Analyses were based on 5,000 bootstrapping samples and 90% bias-corrected confidence intervals (CI). A simple slopes analysis was utilized to probe the moderation using the 16th, 50th, and 84th percentiles as conditioning values.

## Results

### Waiting Time and Willingness to Wait for a Person's Favorite Brand

The analysis revealed a significant interaction effect of waiting time and CBE in relation to a person's willingness to wait for their favorite brand (*b* = 0.10, *p* < 0.059, 90% CI = 0.01–0.18, Δ*R*^2^ = 0.03, *p* < 0.059). At a low level of CBE (point estimate: 16.84) and an average level of CBE (point estimate: 20.00), waiting time (recoded as −1 = 1 month; 1 = 6 months) influenced a person's willingness to wait for their favorite brand (b = −1.16, *p* < 0.001, 90% CI = −1.55 to −0.78 and *b* = −0.86, *p* < 0.001, 90% CI = −1.15 to −0.58, respectively). A longer waiting time diminishes the willingness to wait for a product of their favorite brand. At a high level of CBE (point estimate: 24.00), the waiting time did not influence a person's willingness to wait for their favorite brand (b = −0.48, *p* < 0.068, 90% CI = −0.92 to −0.05). These results are consistent with Hypothesis 2, because the effect of waiting time and willingness of a person to wait for their favorite brand depends on their level of CBE.

## Discussion

Although the literature discusses numerous scales of brand engagement in the Internet behavior context, where indicators refer to social media or online brand community behaviors, brand engagement measurement beyond this context is limited. The aim of the paper was to advance extant research on CBE measurement by presenting a theoretically grounded and empirically validated measure for this construct beyond the Internet behavior context. The application of SET (Emerson, 1976) enables researchers to understand the psychological character of CBE and explore its multifaceted nature. The results of Study 1, which are based on both quantitative and qualitative data, provided a new scale for CBE measurement. Higher-order CFA revealed that the dimensions of CBE constitute a second-order construct. The results of Study 1 and Study 3 provided evidence of the managerial value of the scale, which consists of six items with adequate construct validity (both convergent and discriminant), internal consistency and test-retest reliability. Moreover, scores on the CBE scale predict important variables that are related to consumer marketplace behavior (consumer satisfaction with product and perceived value of brand) and affect the indicator of brand loyalty (Table 4). The use of a diverse sample of stimuli (brands and products) ensured the ecological validity of the research.

**Table 4:** Summary of hypothesis testing.

**Hypothesis**	**Result**	**Findings**
H.1	Supported	CBE is positively related to loyalty intention
H.2	Supported	CBE moderates the relationship between waiting time and willingness to wait for a person's favorite brand: in low-CBE individuals, longer waiting times reduce the willingness to wait for a product of the favorite brand, whereas in high-CBE individuals the willingness to wait does not change depending on the waiting time
H.3	Supported	CBE is positively related to consumer satisfaction with the product
H.4	Supported	CBE is positively related to the perceived value of the brand

### Theoretical Implications

The results of the study have important theoretical implications for the CBE literature. First, previous research on development of CBE scales (e.g., Baldus et al., 2015; Dessart et al., 2016; Schivinski et al., 2016; Harrigan et al., 2017) provided a very useful contribution but concentrated mainly on the Internet behavior aspects of this construct. Consistent with calls for further empirical research on CBE measurement (Islam and Rahman, 2016), this paper adopted the definition of CBE proposed by Hollebeek et al. (2014) and focused on providing a deeper insight into understanding the nature of CBE beyond the Internet behavior context. Consumers' natural descriptions regarding brand engagement behavior derived from structured face-to-face individual interviews confirmed that CBE should be conceptualized as a three-dimensional construct (Hollebeek et al., 2014). Consumers pointed a wide range of CBE indicators. However, only some of these indicators met the measurement criteria in the quantitative study. This means that the indicators included in the final version of the scale refer to the most important aspects of CBE. Other indicators extracted from the qualitative study may be more sensitive to the product context (may be more appropriate for some products than others). It is worth emphasizing that in three studies concerning scale development, I used a broad sample of subjects (product categories and brands) that enabled to draw more general conclusions and suggests that the CBE scale may be applied in diverse product contexts. The CBE scale provides a foundation for building future knowledge on CBE beyond the Internet behavior context by exploring its determinants and consequences.

The proposed CBE scale differs from other measures of offline brand engagement in a substantial way. Namely, none of the four previous scales captures CBE using cognitive, emotional, and behavioral dimensions (So et al., 2014; Vivek et al., 2014; Dwivedi, 2015; Dwivedi et al., 2016). For example, the brand engagement behaviors scale (Dwivedi et al., 2016) focuses only on the behavioral aspect of brand engagement, while the scale of generalized customer engagement (Vivek et al., 2014) captures CBE in terms of cognitive (Conscious Attention) and behavioral (Enthused Participation) dimensions. Apart from the mentioned dimensions, the generalized customer engagement scale includes social dimension (Social Connection), which is not presented in CBE scale proposed in this paper. Social dimension is also included in the scale of customer engagement with tourism brands (So et al., 2014). Although this dimension is not the most frequently analyzed so far, it is gaining in importance especially due to the increasing role of social media in building consumer - brand relationships (Rosado-Pinto and Loureiro, 2020). The CBE scale proposed in this paper has the similar factor structure to the scale developed by Dwivedi (2015). Each of the dimensions of Vigor, Dedication and Absorption (Dwivedi, 2015) corresponds to behavioral, emotional and cognitive dimensions of CBE. Nevertheless, the meaning of these dimensions is slightly different than that proposed in the model of CBE by Hollebeek et al. (2014). Conceptualization of CBE suggested by Dwivedi (2015) is derived from the domain of organizational psychology (work engagement construct). Thus, the understanding of these dimensions is strongly grounded in the work context. Moreover, the CBE scale developed here, contrary to the previous scales, may be employed in diverse brand and product contexts. Whereas, previous scales were validated on single product categories and even with single brands (So et al., 2014; Vivek et al., 2014; Dwivedi, 2015; Dwivedi et al., 2016), the proposed CBE scale was validated using various products and brands.

Second, this paper tested the nomological framework that places CBE as an antecedent of loyalty intention, consumer satisfaction with product, and perceived value of brand. The findings support social exchange theory (Emerson, 1976), which highlights reward-based interaction. Consumers who devote cognitive, affective, and behavioral resources to engaging with brands receive reward in the form of greater satisfaction from a brand (Foa and Foa, 2012). Thus, the research confirmed that the use of social exchange theory as a lens through which CBE is investigated is adequate.

Third, the research was conducted in the developed market context of Poland. The majority of previous research on diverse consumer–brand relationships originated in the U.S., Australia and New Zealand (Islam and Rahman, 2016). Given that variables relating to CBE represent culturally sensitive constructs (Razmus et al., 2020), it is necessary to analyze their measures and test the nomological framework in diverse cultural contexts. Therefore, this paper offers an important addition to the literature by showing that CBE has an important role in explaining consumer behavior variables in the European cultural context and contributes to the broader generalizability of study relationships.

### Managerial Implications

Marketers should know how to encourage consumers to engage in relationships with brands, not only in social media but also beyond the Internet behavior context. The results are helpful in this regard because, based on consumers' natural descriptions concerning brand engagement behavior, they provided a deeper understanding of CBE and its indicators beyond the Internet behavior context. Earlier, practitioners were dependent on measures and indicators of CBE limited mainly to the online environment. While these scales are very useful, brand engagement indicators have limited their use in other contexts. The present research goes beyond this limitation. Therefore, this paper offers a framework that can be used to address the relationship between consumers and brands in real-life settings. Examples of consumers' natural descriptions concerning brand engagement showed that CBE goes beyond the “like” on Facebook. Consumers engage with brand by, for example, feeling of joy in having and using the products of one's favorite brand or defending the brand when others criticize it. These findings can be used by practitioners in improving the management of brands in real-life settings.

The nomological networks of relationships tested in Study 1 indicated that CBE is a promising construct with high relevance to consumer marketplace behavior variables. These findings are consistent with previous studies that showed how constructs related to brand engagement affect consumers' loyalty, satisfaction, brand equity and other marketing variables (Sprott et al., 2009; Schivinski et al., 2016; Carvalho and Fernandes, 2018). The insights from the studies emphasize the importance of managing consumer-brand relationships, including CBE. These findings can be used by marketers in market segmentation processes. Identification of highly engaged consumers who, as shown in this and previous research (Dwivedi, 2015; Islam and Rahman, 2016; Harrigan et al., 2017), are more loyal, can be an important element in creating a marketing offer.

### Limitations and Future Research

This study needs to be interpreted in the context of its limitations. First, the research evaluates the scale's nomological validity only by analyzing the relationship between CBE and three variables: loyalty intention, consumer satisfaction with product and perceived value of brand. Future research should test a wider nomological network and take into account various antecedents and consequences of CBE. Second, the study was based on data from convenience samples, which is typical of scientific research but has limitations in terms of representation and bias. Thus, replicating the psychometric properties of the CBE scale by using a representative sample and examining its cross-cultural validity with data from other countries is warranted. Future research should also analyze how the CBE scale developed in this study differs from the scales of other similar constructs such as brand attitude or brand love. Third, correlational studies had limitations prevalent to the nature of the cross-sectional design. As CBE is a dynamic phenomenon and should evolve over time, building on this idea to gain deeper insight into this dynamic model would be an interesting research direction.

Despite the psychological nature of CBE, this phenomenon has not been given sufficient attention in psychology itself so far (Fetscherin and Heinrich, 2015). Future research could expand the scope of my investigation by studying psychological antecedents and consequences of CBE. For example, scholars can test the role of CBE in experiencing positive product-evoked emotions over diverse stages of a purchase process (Richins, 2013). It is reasonable to assume that CBE would be positively associated with positive product-evoked emotions such as joy and excitement at diverse stages of a purchase process. Future research might also examine the role of CBE in the perception of luxury brands users (Nelissen and Meijers, 2011). It could be expected that consumers with a high level of CBE should perceive people with visible exclusive brand logo more positively.

## Data Availability Statement

The raw data supporting the conclusions of this article will be made available by the authors, without undue reservation.

## Ethics Statement

The studies involving human participants were reviewed and approved by the Research Ethics Committee at the Institute of Psychology at The John Paul II Catholic University of Lublin. Written informed consent for participation was not required for this study in accordance with the national legislation and the institutional requirements.

## Author Contributions

WR: conceptualization, methodology, statistical analysis and investigation, writing-original draft preparation.

## Funding

This work was supported by The National Science Center, Poland, Grant No. DEC- 2018/02/X/HS4/01225.

## Conflict of Interest

The author declares that the research was conducted in the absence of any commercial or financial relationships that could be construed as a potential conflict of interest.

## Publisher's Note

All claims expressed in this article are solely those of the authors and do not necessarily represent those of their affiliated organizations, or those of the publisher, the editors and the reviewers. Any product that may be evaluated in this article, or claim that may be made by its manufacturer, is not guaranteed or endorsed by the publisher.

## Appendix

**Table A1:** Brand engagement indicators and examples of consumer statements.

**Brand engagement indicators**	**Informant**	**Respondent's statements**
**Cognitive**		
1. Positive memories about the brand	man, 27	When I first met my wife I was using Old Spice antiperspirant and I was complimented on that (Old Spice)
2. Thinking about the brand	man, 24	My brand is certainly on my mind all the time; when I purchase a product of a particular brand, my first thought is about buying a product of the brand I have chosen. Other brands simply do not matter to me; I have not devoted time to them and I am not interested in them (Alvarez)
3. Treating the brand as one's own	woman, 26	[…] because my favorite brand is Bioware company, which produces computer games (Bioware)
4. Paying attention to the message related to the brand	man, 29	When I see the message of my favorite brand I always feel engaged in it and I always read it. But I don't usually invest my time in something I feel indifferent about (Wółczanka)
5. Following novelties related to the brand	man, 35	I certainly follow the news about what new products of this brand are launched, about what's new, and about the plans of the brand for the future. (Apple)
**Emotional**		
1. Sense of pride in having and using the products of one's favorite brand	man, 24	It makes me proud and happy to use the services of this brand (Alvarez)
2. Feeling of pleasure in having and using the products of one's favorite brand	man, 27	There certainly is a feeling of pleasure in using, because I like brands that are simply pleasant for me to use (Garnier)
3. Feeling of joy in having and using the products of one's favorite brand	woman, 18	As for emotions, I feel joy; I like this brand very much; I don't like others, and I don't become attached to them as I do to Revlon (Revlon)
4. Lack of strong negative emotions when buying a defective product from this brand	man, 35	[…] I think this won't bother me very much, because I know the company is large and reliable and it will certainly solve this problem somehow for both parties to be satisfied. Of course, I would report it and wait for this issue to be resolved (Apple)
5. Feeling negative emotions in a situation in which the consumer cannot purchase the product of his or her favorite brand	woman	I feel puzzled, because I'm angry if I like a particular thing of this brand but can't have it (H&M)
6. Feeling negative emotions when someone criticizes their favorite brand	man, 43	I'm certainly annoyed. It is not pleasant for me to listen to something like this. It makes me feel as if someone was criticizing my wife (Coldt)
**Behavioral**		
1. The need to advertise the brand by visibly using it	man, 18	I wear Nike shoes when I walk, exercise, or use equipment. In a way, this is also a kind of advertising (Nike)
2. Taking extra effort to obtain the product of one's favorite brand	woman, 21	It was shampoos and hair conditioners. They were rather poorly available in Lublin. My engagement manifested itself in the fact that I was prepared to visit every possible outlet in Lublin where they were available the most often in order to find them (Equilibra)
3. Following information about the brand actively	man, 38	Here's what I do: I follow discussion forums and browse the Web in search of interesting things related to my favorite brand. (Audi)
4. A tendency to recommend the brand to friends	woman, 27	[…] a friend of mine was selling a car recently, so I spent a good half an hour arguing with him and bombarding him with arguments for buying an Opel rather than any other car. (Opel)
5. Defending the brand when others criticize it	man, 22	When someone criticizes Apple and has no sound arguments, I try to logically talk them out of their wrong thinking (Apple)
6. Talking about the brand with others	man, 18	When I am in the company of friends and acquaintances and when a topic somehow related to this brand is brought up, I do praise the brand, because it gives you well-tested quality at a reasonable price. (Vistula)
